# Associations of Serum ALB, IL-18, and NGAL Levels and Individualized Nutritional Nursing in Patients with Hematopoietic Stem Cell Transplantation and Myelosuppression

**DOI:** 10.12669/pjms.42.9.16306

**Published:** 2026-09

**Authors:** Yu Wu, Lei Geng

**Affiliations:** 1 Yu Wu, Department of Hematology, The First Affiliated Hospital of Soochow University, Suzhou, Jiangsu Province 215000, P.R. China; 2 Lei Geng, Department of Hematology, The First Affiliated Hospital of Soochow University, Suzhou, Jiangsu Province 215000, P.R. China

**Keywords:** Albumin, Hematopoietic Stem Cell Transplantation, Interleukin-18, Individualized Nutritional Intervention and Nursing, Neutrophil Gelatinase-Associated Lipocalin

## Abstract

**Objective:**

To investigate the clinical associations of serum albumin (ALB), interleukin-18 (IL-18), and neutrophil gelatinase-associated lipocalin (NGAL) and individualized nutritional nursing on the recovery of patients in the myelosuppression stage of the conditioning regimen of hematopoietic stem cell transplantation (HSCT).

**Methodology:**

Retrospectively selecting clinical data of 118 patients undergoing hematopoietic stem cell transplantation First Affiliated Hospital of Soochow University, Department of Hematology, from January 2024 to September 2025, 56 patients receiving routine care from January 2024 to November 2024 were selected as the control group, and 62 patients receiving personalized nutritional nursing from December 2024 to September 2025 were selected as the study group. Dynamics of serum ALB, IL-18, and NGAL were tested. Statistics on hematopoietic function reconstruction, serological indicators, complications, and hospitalization-related indicators during the bone marrow suppression period in two groups.

**Results:**

The study group had lower neutrophil implantation time, platelet implantation time, red blood cell transfusion volume, and platelet transfusion volume compared to the control group (P<0.05). Serum levels of ALB in the study group were enhanced, while serum levels of IL-18 and NGAL were reduced compared with those in the control group (P<0.05). The incidence of complications in the study group (9.68%) was lower than that in the control group (21.43%), but there was no significant difference between the groups (P>0.05). The duration of bone marrow suppression and total hospitalization time in the study group were shorter than those in the control group, but there was no significant difference between the groups (P>0.05).

**Conclusion:**

Individualized nutritional nursing can accelerate the reconstruction of bone marrow hematopoietic function and may reduce the incidence of complications, and shorten the length of hospital stay, potentially through modulation of serum ALB, IL-18, and NGAL levels during the conditioning regimen of HSCT.

## INTRODUCTION

As a critical pre-hematopoietic stem cell transplantation (HSCT) procedure, the conditioning regimen involves high-dose radiotherapy and chemotherapy to eliminate abnormal cells, suppress immune responses, and create favorable conditions for hematopoietic stem cell engraftment.^1^,^2^ However, the high-intensity conditioning regimen is associated with severe adverse effects. It inevitably causes tissue and metabolic damage, induces severe myelosuppression, leads to complications such as agranulocytosis, thrombocytopenia, and anemia, increases life-threatening complications, including infection and hemorrhage, and directly affects the success rate of transplantation and patient prognosis.^3^,^4^ The nutritional status is widely recognized as an independent risk factor that influences quality of life and tolerance to the proposed treatment.^5^ Artificial nutrition, especially total parenteral nutrition, is administered to patients receiving HSCT to reduce negative nutritional effects.^6^ Although those patients benefit from nutritional therapy, wide variations regarding the start and stop time points of nutritional therapy as well as route of delivery have been found in patients undergoing HSCT.^7^ Identifying and minimizing barriers is necessary to enhance current practices and provide high-quality treatment.

Clinically, multiple laboratory biochemical indicators, such as albumin (ALB), transthyretin, transferrin, and retinol-binding protein, have been used as simple, objective tools for assessing the nutritional status of patients.^8^ Among them, serum albumin (ALB) is a core indicator reflecting the body’s nutritional status and inflammatory stress level. A decrease in its level usually indicates insufficient nutritional reserves and impaired immune function in patients.^9^ Additionally, inflammatory mediators have been recognized as potential biomarkers reflecting the nutritional status of patients. Chronic low-grade inflammation is associated with altered nutritional status in patients with high body mass index (BMI)^10^, Type-2 diabetes^11^, cancer^12^, and cardiovascular diseases.^13^ Interleukin-18 (IL-18), an important pro-inflammatory cytokine involved in Th1 responses and regulation of immunity, is highly expressed in tissue damage and inflammatory responses induced by the conditioning regimen.^14^ In a murine model with immune-mediated bone marrow (BM) failure, IL-18 levels in the plasma were significantly enhanced. Higher levels of IL-18 were detected in patients with acquired aplastic anemia, a disease characterized by bone marrow failure.^15^ Moreover, IL-18 is closely correlated with the degree of bone marrow hematopoietic function inhibition.^16^ Neutrophil gelatinase-associated lipocalin (NGAL), which is also known as lipocalin 2, is a glycoprotein that was originally purified and characterized from the granules of human neutrophils.^17^ NGAL is elevated in the bone marrow of patients with hematologic malignancy^18^, and its plasma levels have a significant association with iron status in anemic patients with chronic kidney disease.^19^ Notably, NGAL can sensitively reflect the damage and recovery process of hematopoietic tissues, and is considered a potential biomarker for evaluating the reconstruction of bone marrow hematopoietic function.^20^–^22^

At present, most clinical nutritional interventions for patients during the HSCT conditioning period use standardized protocols that do not assess levels of metabolic indicators and inflammatory factors and lack individualized adjustments, making it difficult to accurately meet patients’ nutritional needs and reduce myelosuppression.^23^ This study retrospectively evaluated the associations between serum ALB, IL-18, and NGAL levels, individualized nutritional nursing, and clinical outcomes in patients undergoing HSCT during the myelosuppression stage. It also explored the impact of individualized nutritional nursing on hematopoietic recovery and the risk of complications. The results may provide evidence to optimize nursing strategies for HSCT patients and improve transplantation outcomes.

## METHODOLOGY

Retrospectively selecting clinical data of 152 patients undergoing hematopoietic stem cell transplantation First Affiliated Hospital of Soochow University, Department of Hematology, from January 2024 to September 2025. Those patients were selected based on inclusion criteria and exclusion and 118 patients were finally included. Based on strategies of nutritional support, 56 patients receiving routine care from January 2024 to November 2024 were allocated to the control group, and 62 patients receiving personalized nutritional intervention nursing from December 2024 to September 2025 were allocated to the study group. Statistics on receiving serum levels of ALB, IL-18, and NGAL, hematopoietic function reconstruction, serological indicators, complications, and hospitalization-related indicators during the bone marrow suppression period in the two groups.

### Ethical approval:

The ethics committee of our hospital approved this study with the number: 2025882; dated September 25, 2025.

### Inclusion criteria:

- Patients diagnosed with malignant hematological diseases^24^ or severe aplastic anemia^25^ who underwent HSCT for the first time
- Aged 18–65 years, with body mass index (BMI) ranging from 18.0 to 25.5 kg/m² and an expected survival time of ≥ 6 months.
- Basically normal liver and kidney functions as well as coagulation function before the conditioning regimen.
- Karnofsky Performance Status (KPS) score ≥ 70 points before transplantation.
- Complete clinical data.

### Exclusion Criteria:

- Patients with malignant tumors at other sites, severe autoimmune diseases or organ failure.
- Patients with severe malnutrition, gastrointestinal bleeding or malabsorption before the conditioning regimen.
- Patients with a history of previous HSCT, or contraindications to lumbar puncture or bone marrow puncture.
- Pregnant or lactating women.
- Patients with incomplete clinical data.

### Basic conditioning regimen:

Interventions were initiated on the first day of the conditioning regimen for all patients. The intervention cycle lasted until the reconstruction of hematopoietic function during the myelosuppression stage (absolute neutrophil count ≥ 0.5×10^9^/L, platelet count ≥ 20×10^9^/L). During this period, all patients received basic medical measures for hematopoietic stem cell transplantation (HSCT), including standardized conditioning chemotherapy, stem cell infusion, anti-infection treatment, and symptomatic supportive medication.

### Routine nursing care

### Aseptic Management:

Disinfection and isolation measures in laminar flow wards were strictly implemented. Air and surface disinfection were performed twice daily. Patients were instructed to maintain oral, perianal, and skin hygiene to prevent infection.

### Disease Monitoring:

Vital signs such as body temperature, heart rate, and blood pressure were monitored daily. Routine blood tests and liver and kidney function tests were conducted regularly. Gastrointestinal reactions (e.g., nausea, vomiting, diarrhea) and mucocutaneous bleeding in patients were recorded and reported to physicians for timely management.

### Psychological care:

Patients and their family members were informed of the transplantation process and precautions during the myelosuppression stage to alleviate anxiety and fear. Patients were guided to perform bed-based limb exercises to prevent deep vein thrombosis.

### Standardized nutritional support:

A fixed nutritional protocol was formulated, with a daily caloric supply of 30–35 kcal/kg and protein supply of 1.0–1.2 g/kg. Priority was given to oral intake of high-protein, high-calorie, and easily digestible diets (e.g., steamed egg custard, fish puree, nutritious porridge). If patients developed anorexia, nausea, or vomiting, the diet was adjusted to a liquid or semi-liquid form. When necessary, enteral nutritional preparations (e.g., Fresubin, Supportan, Total Protein [TP] Enteral Nutrition Powder, Nutrison, Peptisorb, Total Nutrient Admixture [TNA]) were administered via nasogastric tube pump at a dose of 500–1000 mL/d according to patient tolerance. In cases of insufficient enteral nutrition, a compound amino acid injection was administered via intravenous infusion.

### Personalized nutritional intervention nursing

A combined regimen of serum ALB, IL-18, and NGAL detection plus individualized nutritional intervention and nursing care included the following:

### Dynamic Detection of Serological Indicators and Three-level Nutritional Diagnosis

### Serological Indicator Detection:

Fasting cubital venous blood samples (5 mL each) were collected from patients on the Preprocess the 1st and 4th days of the conditioning regimen in the early morning. Serum ALB level was measured by the bromocresol green method (Cat: IB6680, Solarbio, Beijing, China), IL-18 level was determined by enzyme-linked immunosorbent assay (ELISA) (Cat: SEKH-0028, Solarbio, Beijing, China), and NGAL level was measured by immunoturbidimetry (Cat: SEKH-0267, Solarbio, Beijing, China). Within 24 hours, test results were reported back to the multidisciplinary nutritional intervention team, which consisted of hematologists, specialized nutrition nurses, clinical dietitians, and primary nurses.

### Three-level Nutritional Diagnosis Process:

Within 24 hours of admission, primary nurses performed nutritional screening (Level-1 diagnosis) using the Nutritional Risk Screening 2002 (NRS2002) scale. A score ≥ 3 indicated high nutritional risk. Within 48 hours of admission, nutrition nurses and clinical dietitians conducted a nutritional assessment (Level 2 diagnosis) for high-risk patients using the Patient-Generated Subjective Global Assessment (PG-SGA) scale to classify malnutrition severity as mild, moderate, or severe. Within 72 hours of admission, the multidisciplinary team completed a comprehensive evaluation (Level 3 diagnosis), including medical history collection, physical examination, serological indicator testing, and instrumental examinations, to identify the type and cause of malnutrition.

### Quantitative Basic Nutrient Supply Protocol:

To maintain patients’ ideal body weight, the basic protocol was formulated in accordance with nutrient supply standards for cancer patients:

### Energy:

For patients without severe complications, energy supply was set at 25–30 kcal/(kg·d); for patients with moderate to severe malnutrition or complicated with infection, the supply was increased to 35 kcal/(kg·d).

### Protein:

The basic protein supply was 1.2–1.5 g/(kg·d), accounting for 15%–20% of total energy intake, with high-quality protein (e.g., whey protein, fish) accounting for ≥ 50% of the total protein. For protein-deficient patients (ALB < 35 g/L), the supply was increased to 1.5–2.0 g/(kg·d).

### Fat:

Fat accounted for 25%–30% of total energy intake, with priority given to unsaturated fatty acids (e.g., olive oil, deep-sea fish oil).

### Carbohydrates:

Carbohydrates accounted for 50%–60% of total energy intake, and low-glycemic-index foods such as whole grains and mixed beans were preferred.

Stepwise Nutritional Intervention Based on Intake Stratification and Serological Indicators Combined with the ratio of patients’ actual nutrient intake to the normal requirement (assessed via the 24 hours dietary recall method). Intervention measures were adjusted in accordance with the stepwise nutritional support principles specified in the 2025 Expert Consensus on Nutritional Intervention for Major Digestive System-related Nutritional Impact Symptoms in Cancer Patients, as well as the results of serological indicators: 1) Oral nutritional supplements (ONS) of total protein (TP) enteral nutrition powder (Ensure), six scoops per time, three times a day, were administered. Strategies were made to ensure the serum ALB level to reach ≥ 35 g/L. 2) IL-18 > 450 pg/mL indicates intestinal mucosal inflammation^26^,^27^: The diet was adjusted to a low-residue, non-irritating liquid diet (heated in a microwave oven). If nutrient intake was < 50% of the normal requirement, tube feeding (TF) enteral nutrition was provided as a supplement. 3) NGAL > 150 ng/mL indicates hematopoietic tissue damage^28^-^29^: Daily intake of 50–100 g of microwave-heated animal liver or duck blood was recommended to supplement heme iron. For patients with poor appetite whose nutrient intake was < 75% of the normal requirement, high-energy, high-protein ONS was used for reinforcement; if oral administration was intolerable (intake < 25% of the normal requirement), parenteral nutrition (PN) with a total nutrient admixture (TNA) was adopted.

### Efficacy Evaluation and Protocol Maintenance:

After each round of serological testing, the efficacy of the intervention was evaluated based on patients’ body weight, nutrient intake, and clinical symptoms. When the indicators returned to normal for two consecutive tests and nutrient intake reached ≥ 75% of the normal requirement, the regimen was adjusted to a maintenance nutritional protocol with protein supply of 1.2–1.5 g/(kg·d) until the patients were discharged from the hospital.

### Observation Indicators:

- The collected indicators included:
- Hematopoietic function reconstruction during the myelosuppression stage, including the time to neutrophil engraftment (the interval from hematopoietic stem cell infusion to an absolute neutrophil count ≥ 0.5×10^9^/L), time to platelet engraftment (the interval from hematopoietic stem cell infusion to a platelet count ≥ 20×10^9^/L without the need for platelet transfusion), red blood cell transfusion volume, and platelet transfusion volume.
- Serological indicators, including serum ALB, IL-18, and NGAL.
- Incidence of complications during the myelosuppression stage, including infection (fever accompanied by positive etiological findings or clinically diagnosed infection), hemorrhage (mucocutaneous hemorrhage, gastrointestinal hemorrhage, etc.), oral ulcers, diarrhea, and liver and kidney function impairment.
- Hospitalization-related indicators, including the duration of the myelosuppression stage and total length of hospital stay.

### Statistical analysis:

The data were analyzed using SPSS 25.0. Continuous variables with normal distribution were expressed as mean ± standard deviation (\bar{x}\pm s) and compared using the independent-samples Student’s t-test. For non-normally distributed variables, the Mann-Whitney U statistic was performed. For categorical variables, they were described as frequencies and constituent ratios (%). Chi-square test was carried out when sample sizes were over five, while Fisher’s exact test was used when expected frequencies were small. A value of P < 0.05 was considered statistically significant.

## RESULTS

As shown in Table-I, there were no statistically significant differences in baseline data, including age, gender, BMI, disease type, transplantation type, conditioning regimen, and disease course, between the two groups (P>0.05), indicating good comparability. Compared with the control group, the study group had significantly shorter times to neutrophil and platelet engraftment, as well as lower red blood cell and platelet transfusion volumes (P < 0.05; Table-II).

**Table-I:** Comparison of General Data Between the Two Groups.

Items	Study Group(n=62)	Control Group(n=56)	t/χ^2^value	P value
Age (*x̄*±*s*, years)	41.26±10.35	40.89±10.12	0.196	0.845
Gender[n(%)]				
Male	34(54.84)	27(48.21)	0.517	0.472
Female	28(45.16)	29(51.79)		
Body Mass Index (*x̄*±*s*, kg/m^2^)	21.86±1.67	22.05±1.54	0.640	0.523
Disease Type[n(%)]				
Acute Myeloid Leukemia	28(45.16)	19(33.93)	2.148	0.342
Acute Lymphoblastic Leukemia	19(30.65)	24(42.86)		
Myelodysplastic Syndrome	15(24.19)	13(23.21)		
Transplantation Type[n(%)]				
Autologous Hematopoietic Stem Cell Transplantation	38(61.29)	29(51.79)	1.083	0.298
Allogeneic Hematopoietic Stem Cell Transplantation	24(38.71)	27(48.21)		
Conditioning Regimen[n(%)]				
Modified BuCy Regimen	36(58.06)	26(46.43)	0.469	0.494
TBI+Cy Regimen	26(41.94)	30(53.57)		
Disease Course(*x̄*±*s*, months)	9.25±3.12	8.98±3.45	0.446	0.656

***Note:*** BuCy: Busulfan-Cyclophosphamide; TBI: total body irradiation; Cy: cyclophosphamide.

**Table-II:** Comparison of Hematopoietic Function Reconstruction Indicators during Myelosuppression between the Two Groups (*x̄*±*s*).

Group	Case Number	Time to Neutrophil Engraftment (d)	Time to Platelet Engraftment (d)	Red Blood Cell Transfusion Volume (U)	Platelet Transfusion Volume (U)
Study Group	62	11.25±2.13	13.56±2.87	2.13±0.85	3.26±1.12
Control Group	56	14.89±2.56	16.65±3.12	3.07±1.02	5.89±1.34
*t* value		8.424	5.604	5.456	11.606
*P* value		<0.001	<0.001	<0.001	<0.001

As shown in Table-III, on the preprocessing day one, there were no significant differences in ALB, IL-18, and NGAL levels between the two groups (P > 0.05). On the preprocessing day four, ALB levels were significantly higher, whereas IL-18 and NGAL levels were significantly lower in the study group than in the control group (all P<0.001). Table-IV shows that the incidence of complications in the study group (9.68%) seemed to be lower than that in the control group (21.43%), but no significant difference was achieved (χ²=3.143, P=0.076). The duration of myelosuppression and total length of hospital stay were shorter in the study group, but the differences were not statistically significant (P>0.05; Table-V).

**Table-III:** Comparison of Serological Indicator Levels Between the Two Groups(*x̄*±*s*).

Time	Group	Case Number	ALB (g/L)	IL-18 (pg/mL)	NGAL (ng/mL)
Preprocessing day 1	Study Group	62	32.15±2.34	520.34±65.89	182.66±30.12
	Control Group	56	31.78±2.02	515.67±62.54	179.98±28.56
	*t value*		0.915	0.394	0.495
	*P value*		0.362	0.694	0.622
Preprocessing day 4	Study Group	62	38.67±2.56	310.11±45.67	111.56±21.34
	Control Group	56	35.54±2.45	344.78±50.12	135.09±23.65
	*t value*		6.768	3.932	5.682
	*P value*		<0.001	<0.001	<0.001

**Table-IV:** Comparison of the Incidence of Complications During Myelosuppression Between the Two Groups [n(%)].

Group	Case Number	Infection	Hemorrhage	Oral Ulcer	Diarrhea	Hepatic and Renal Function Impairment	Total Incidence
Study Group	62	1(1.61)	0(0.00)	3(4.84)	1(1.61)	1(1.61)	6(9.68)
Control Group	56	4(7.14)	4(7.14)	1(1.79)	0(0.00)	3(5.36)	12(21.43)
*χ^2^*value							3.143
*P* value							0.076

**Table-V:** Comparison of Hospitalization-related Indicators Between the Two Groups(*x̄*±*s*).

Group	Case Number	Duration of Myelosuppression (d)	Total Length of Hospital Stay (d)
Study Group	62	18.56±6.21	35.53±5.17
Control Group	56	20.34±5.56	37.89±9.32
*t* value		1.634	1.466
*P* value		0.105	0.145

## DISCUSSION

This study demonstrated that monitoring ALB, IL-18, and NGAL levels during the conditioning regimen of HSCT and implementing individualized nutritional interventions are associated with accelerated recovery of bone marrow hematopoietic function. The combined regimen was associated with improved hematopoietic recovery and showed trends toward reduced complications and shorter hospitalization stay.

The high-dose chemoradiotherapy during the conditioning phase of HSCT triggers intense systemic stress responses, leading to damage to the hematopoietic microenvironment, cytokine storm, and nutritional and metabolic disorders, further prolonging the myelosuppression stage, increasing the risk of infection and hemorrhage, and severely affecting prognosis.^30^ Therefore, improving patients’ physical status during the conditioning phase through precise monitoring and individualized intervention has become a core clinical issue for optimizing recovery during the myelosuppression stage. This study innovatively combined dynamic monitoring of serum ALB, IL-18, and NGAL with individualized nutritional intervention, achieving significant clinical effects.

This study showed that ALB levels in patients who were treated with the combined regimen were significantly higher than in the control group, whereas IL-18 and NGAL levels were significantly lower. Additionally, hematopoietic function reconstruction indicators were better in the study group (P < 0.05), consistent with previous research. A previous study has pointed out that decreased serum ALB levels before allogeneic stem cell transplantation are significantly closely associated with non-relapse mortality (p=0.01).^31^ In another retrospective study enrolling 113 patients with lymphoma and treatment with high-dose chemotherapy + autologous stem cell transplantation, those patients with lower ALB levels in the serum before therapy had poorer progression-free survival and overall survival. Therefore, patients with low albumin levels require intensive care and effective maintenance therapy following transplantation.^32^

Accumulated evidence has shown that the excessive activation of inflammasomes precipitate inflammatory damage in acute graft-versus-host disease, an essential complication of HSCT.^33^ IL-1β and IL-18 are two common proinflammatory cytokines produced by activated inflammasomes. Higher serum IL-18 levels before HSCT have been found with significant associations with poorer clinical outcomes after autologous stem cell transplantation.^34^ Our results may provide a rationale for prospective studies evaluating IL-18 status and inhibition of IL-18 activity in patients undergoing allografting. Previous studies have confirmed that elevated IL-18 levels during the conditioning phase and early post-transplantation are significantly associated with an increased risk of delayed engraftment of neutrophils and platelets.^21^ Those studies suggested that IL-18 levels in the serum might be a potential biomarker of HSCT. Moreover, IL-18 had a significant association with fat-free mass in healthy adults (p=0.03). The fat-free mass compartment, which has been regarded as a well-known confounder in evaluating insulin sensitivity, may significantly contribute to the relationship between IL-18 and insulin action.^35^ Consistent with the results of this study, we also suggested that decreased IL-18 levels are accompanied by accelerated recovery of hematopoietic function.

A study by Benoit SW et al.^36^ confirmed that NGAL levels can serve as an effective marker for the hematopoietic recovery of patients undergoing autologous HSCT. In this study, NGAL levels on the day of hematopoietic reconstruction in the study group were significantly reduced, confirming the promoting effect of individualized nutritional intervention on hematopoietic tissue repair and providing clinical evidence for the application of this indicator in nutritional intervention for HSCT.

A study by Zama D et al.^37^ showed that although enteral nutrition can reduce the incidence of acute graft-versus-host disease, it does not significantly improve hematopoietic reconstitution efficiency, possibly due to the lack of targeted nutritional adjustment strategies. In contrast, this study identified the synergistic effect of nutritional support, inflammatory regulation, and hematopoietic repair by guiding protein supplementation based on ALB levels, adding ω-3 polyunsaturated fatty acids to counter inflammation when IL-18 is highly expressed, and strengthening the supply of hematopoietic nutrients when NGAL is elevated.

The results showed that the time to neutrophil and platelet engraftment in the study group was significantly shortened, and the transfusion volumes of red blood cells and platelets were reduced (P < 0.05), consistent with the research conclusion of Evans JC et al.^38^ Targeted nutritional support can accelerate hematopoietic recovery by improving nutritional status, reducing metabolic damage, and lowering the risk of transfusion-related complications. However, a trial by Oratz T et al.^39^ did not find a significant improvement in quality of life with individualized nutritional intervention. This discrepancy may be related to the fact that the study did not combine indicators of inflammation and hematopoietic injury and had a single intervention target, further highlighting the benefits of the multi-indicator-combined monitoring scheme in this study.

Although the incidence of complications in the study group (n=6, 9.68%) was lower than that in the control group (n=12, 21.43%), this difference was not significant (χ²=3.143, P=0.076). Shoug Alsalamah et al.^40^ have reported that nutritional support for HSCT patients can reduce the incidence of infection, with a risk ratio of 0.72 for intravenous glutamine supplementation. In this study, anti-inflammatory nutritional intervention and nursing guided by inflammatory indicators further enhanced the effect of infection prevention and control. Similarly, our study also suggested that the study group had reduced levels of IL-18 and NGAL following personalized nutritional intervention nursing.

Although the total length of hospital stay was numerically shorter in the study group, the difference did not reach statistical significance (P=0.145). This trend may suggest a potential benefit of individualized nutritional intervention on recovery efficiency; however, larger prospective studies are needed to further verify this finding.^41^ Furthermore, a study by Baena-Gómez MA et al.^42^ showed that ω-3 polyunsaturated fatty acids can inhibit the release of inflammatory factors and reduce gastrointestinal mucosal damage, which may explain the core mechanism for the reduced incidence of gastrointestinal complications such as oral ulcers and diarrhea, observed in this study.

The main innovation of this study lies in the implementation of precision-targeted nutritional intervention and nursing based on the dynamic changes of ALB, IL-18, and NGAL. Before HSCT, determining the levels of the three indicators would offer a distinct advantage over traditional standardized nutritional support. Since there are significant individual differences in the nutritional needs of HSCT patients, nutritional regimens formulated solely based on body weight or fixed formulas are unable to account for the dynamically changing physiological needs of patients.

### Limitations:

It is a single-center retrospective study with a relatively small sample size. A long-term follow-up was not conducted, making it impossible to evaluate the impact of this intervention plan on patients’ long-term quality of life. In addition, the differences in intervention effects among patients with different disease types and transplantation methods were not analyzed in depth.

## CONCLUSIONS

Monitoring ALB, IL-18, and NGAL during the conditioning phase of HSCT and implementing individualized nutritional interventions and nursing care can accelerate the recovery of bone marrow hematopoietic function and may reduce complications, and shorten hospital stay.

### Recommendations:

Future multi-center, prospective, large-sample studies with extended follow-up time are needed to further optimize the intervention plan. Additionally, applying artificial intelligence algorithms to dynamic indicator monitoring and decision-making for intervention can improve the accuracy and timeliness of nutritional interventions.

### Authors’ contributions:

**YW:** Literature search, study design and manuscript writing.

**YW and LG:** Data collection, data analysis and interpretation. Critical review.

**YW:** Manuscript revision and validation and is responsible for the integrity of the study.

All authors have read and approved the final manuscript.
